# Supplementary figures and images for: α-Synuclein seeding activity in duodenum biopsies from Parkinson’s disease patients
Source: PLoS Pathog. 2023 Jun 30;19(6):e1011456. doi: 10.1371/journal.ppat.1011456 (PMC10313076; doi:10.1371/journal.ppat.1011456)

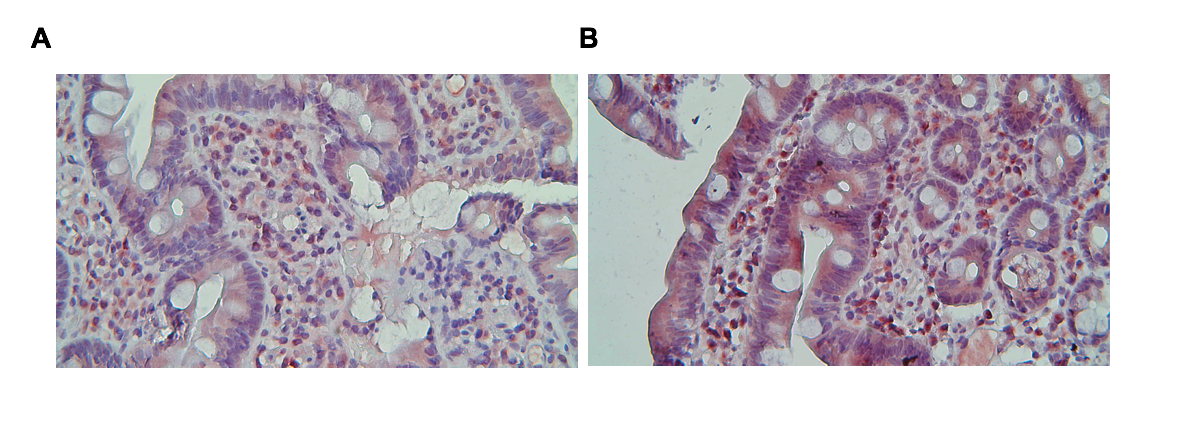

Supplement: S1 Fig — (A) Immunohistochemistry for tyrosine hydroxylase (TH). (B) Immunohistochemistry for choline acetyltransferase (ChAT). (TIF) [file ppat.1011456.s001.tif]

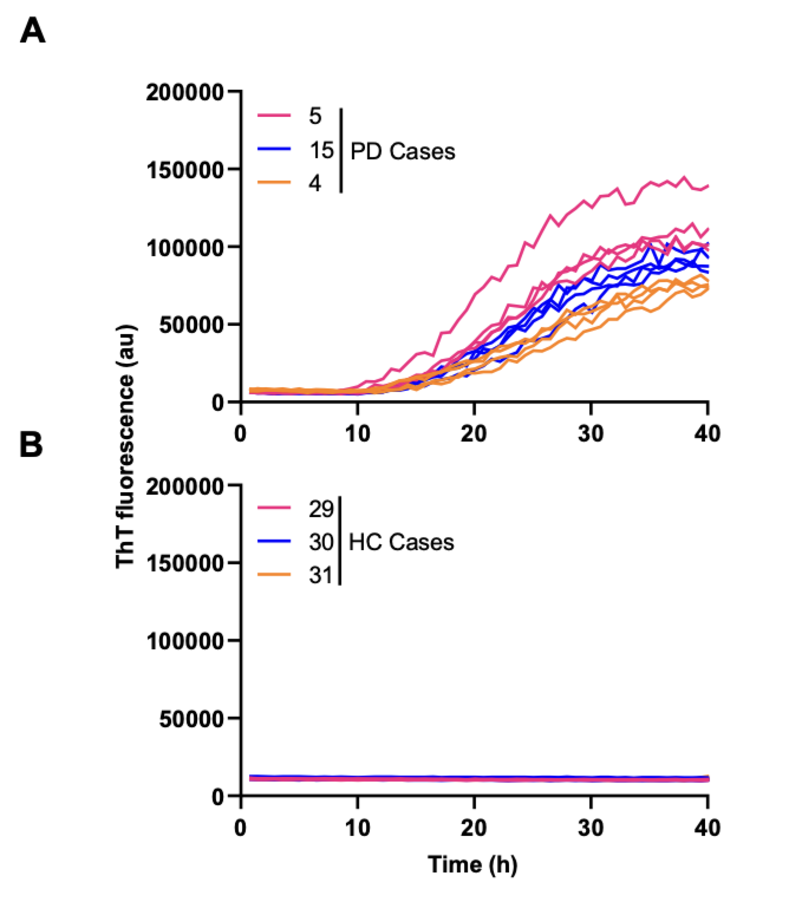

Supplement: S2 Fig — (A) Traces from single reactions (n = 4 per case) seeded with 10−3 dilutions of duodenum IM biopsies from PDs cases 4, 5, and 15 (as colored) as a function of reaction time. (B) Traces from healthy control duodenum IM biopsies from cases 29, 30, and 31. (TIF) [file ppat.1011456.s002.tif]
