# Supplementary material for: α-Synuclein seeding activity in duodenum biopsies from Parkinson’s disease patients
Source: PLoS Pathog. 2023 Jun 30;19(6):e1011456. doi: 10.1371/journal.ppat.1011456 (PMC10313076; doi:10.1371/journal.ppat.1011456)
Supplement: S1 Text — (DOCX) [file ppat.1011456.s004.docx]

**Supporting information**

**Immunohistochemistry analysis of human** **intestinal mucosa biopsies**

Immunohistochemistry for tyrosine hydroxylase (TH) and choline acetyltransferase (ChAT) (S1 Fig) were done as previously described [1]. Briefly, after rehydration of 4 μm thick sections, heat-induced antigen retrieval was performed in 10 mM citrate buffer, followed by incubation in normal goat serum. Sections were then incubated overnight at 4°C with rabbit polyclonal antibodies diluted 1:1000 against TH (AB #152, Millipore, Temecula, California), or ChAT (AB#143, Millipore). Biotin-conjugated goat anti-rabbit (BA-1000, RRID:AB_2313606, Vector, Burlingame), secondary antibody was used. The reaction product was revealed by the ABC (Cat#G011-61, BioSpa Div., Milan, Italy), diluted 1:250, followed by incubation with the NovaRed chromogen.

**Raw traces of the ThT fluorescence curves**

(S2 Fig)

**References**

1. Trucas, M., et al., *The density of hepatic autonomic innervation differs between compensatory and direct hyperplasia rat models.* J Peripher Nerv Syst, 2023. **28**(1): p. 98-107.
