# Supplementary material for: α-Synuclein seeding activity in duodenum biopsies from Parkinson’s disease patients
Source: PLoS Pathog. 2023 Jun 30;19(6):e1011456. doi: 10.1371/journal.ppat.1011456 (PMC10313076; doi:10.1371/journal.ppat.1011456)
Supplement: Attachment — Submitted filename: Rebuttal_Plos Pathogens.pdf [file ppat.1011456.s005.pdf]

Dear Editor,

Thank you for giving us the opportunity to improve our manuscript with the helpful advice of the reviewers as described below.

First, we want to point to a word change in the abstract (“prove” --> “provide evidence”) that was not suggested by the reviewers, but we think is more accurate.

We also have corrected in the author list the symbols indicating “These authors contributed equally to this work” and we have added the initial of middle name for one of the authors (Bradley R. Groveman). The initial of his middle name has been also added in the “Author Contribution“ section, as well as his name in Data curation in the same section.

The panel A and B or C in the legends of Figure 5 and 7, and in Supporting information captions of S1 Figure have been changes in bold and parentheses. We have also corrected “Constipation score” and “Disease duration” in refers to panel B and C of Figure 7 legend, respectively. The indication of “S1 Fig” and “Raw traces of the ThT fluorescence curves” related to S2 Fig have included in the Supporting information file.

The reference list has been altered due to the addition of a reference mentioned by Reviewer #2 in her first minor comment (reference 10 on lines 64 and 255). Other four references have been added in the revised sentences on line 83 (references 18 and 26) and on line 247 (references 19 and 25).

Reviewer's Responses to Questions

Part I – Summary

Reviewer #1: In this manuscript, Vascellari and colleagues investigate αSyn seeding activity in duodenal biopsies from PD and control patients using a modified version of RT-QuIC called RT-QuICR. They find positive seeding activity in 22/23 PD patients compared to none of the 6 control samples investigated. These results suggest that RT-QuICR-mediated detection of αSyn seeding activity in intestinal biopsies may be useful as an ante mortem diagnostic test for PD.

This is a well-conducted set of experiments on an important topic. The conclusions are sound and the manuscript is well written. I was left with an overall positive impression of the manuscript with only relatively minor issues for the authors to address.

>>We thank the reviewer for their supportive comments.

Reviewer #2: The authors performed asyn- (and tau) seeding assay on duodenal biopsies of PD patients and healthy controls. Despite the authors did not observe any correlation of duodenal asyn levels with clinical parameters, I find the findings highly interesting and relevant to the field, esp. in light of progress towards earlier diagnosis which is currently lacking. The manuscript is very well-written, figures are clear and the methods are executed adequate including quadruplicates which is highly recommended when using RT-QuIC.
I only have a few minor comments to the discussion.

>>We thank the reviewer for her supportive comments.

Reviewer #3: In the manuscript ‘α-synuclein seeding activity in duodenum biopsies from Parkinson’s disease patients,’ the authors find that using the RT-QUIC rapid assays they are able to detect α-synuclein seeding activity. Uniquely the authors can discover α-synuclein end-point dilution seeding units per mg of tissue, allowing for possible future diagnostics. This manuscript adds to the field of understanding how misfolded proteins, like α-synuclein accumulates in the gut and should be considered for publication after the following minor concerns.

>>We thank the reviewer for her supportive comments.

Part II – Major Issues: Key Experiments Required for Acceptance

Reviewer #1: 1. The authors mention that their RT-QuICR results are in agreement with previous results showing αSyn pathology in the duodenum (Emmi et al., 2023). However, this does not address the previous inconsistencies in IHC data the authors note in the introduction (Corbille et al., 2016; Emmi et al., 2023; Ruffmann et al., 2018). Inclusion of IHC for αSyn in the current patient duodenal biopsy samples would be a stronger comparison and would address the important question of whether RT-QuICR is always in agreement with IHC data or does it show increased sensitivity that may explain previously noted IHC discrepancies (Corbille et al., 2016; Emmi et al., 2023; Ruffmann et al., 2018)?

>>We understand this concern. All precedent suggests that RT-QuIC is considerably more sensitive than IHC, so we would not, a priori, expect IHC to always agree with RT-QuIC. Nonetheless, we agree that it would have been nice, formally, to have been able to provide IHC data on the same biopsies that we tested by RT-QuIC. Unfortunately, these biopsies are rather small, and an initial attempt to perform aSyn IHC on them failed for technical reasons, leaving insufficient tissue to try again. Thus, we are unable to provide IHC data on these biopsies.

2. The average age of the non-PD patients from which the healthy control biopsies were derived is nearly 10 years younger than the PD patient population used (to go along with far fewer samples in the control group). While this reviewer appreciates that there are likely challenges with obtaining sufficient numbers of perfectly aged-matched control duodenal biopsies, the authors need to discuss how the different ages could at least partially influence their findings. For instance, the 3 control samples that produced a positive response in at least one replicate reaction (IM28, IM29, and IM31) in Figures 3 and 5A are from the oldest controls, which have an average age (64.7 yr) that is much closer to the mean age of the PD sample group. Thus, IM RT-QuICR may be less discriminatory between PD and controls as age increases.

>>The reviewer raises a reasonable point. We have now considered this caveat on lines 281-283.

Reviewer #2: (No Response)

Reviewer #3: (No Response)

Part III – Minor Issues: Editorial and Data Presentation Modifications
Please use this section for editorial suggestions as well as relatively minor modifications of existing data that would enhance clarity.

Reviewer #1: 1. As PEG-J for continuous L-DOPA infusion is generally used in more advanced cases of PD where oral L-DOPA is ineffective, is there any concern that this may artificially select for patients with severe PD and that the results may not be generalizable to patients with more moderate/mild PD that can be controlled well via oral L-DOPA?

>>This is an interesting consideration that we now discuss on lines 277-281.

2. No raw traces of the ThT fluorescence curves were included. It would be useful to include some (or all) raw traces, perhaps as a supplementary figure, so that researchers conducting similar studies have examples of what such curves should look like. Were the ThT plateau fluorescence values similar across all positive samples?

>>We agree that it would be helpful to show raw traces, and we have now added representative examples in supporting S2 Fig and Supporting information.

supporting S1 Fig and supporting material
3. The authors mention using "a mutated version of human α-synuclein protein to achieve faster and more specific detection of α-SynD". While K23Q does improve specificity due to reduced propensity for spontaneous aggregation, I question if it results in "faster" detection. Previous work shows K23Q had near identical elongation kinetics to WT αSyn when seeding with WT PFF and in αSyn RT-QuIC (Groveman et al., 2018; Koo et al., 2008).

>>This is a good point. We meant to indicate that the assay was much faster than previous SAAs, but not this was due to the use of K23Q. Accordingly, we have revised the sentence on lines 80-83.

4. In calculating the SD50, several samples did not reach end-point requiring the extrapolation of the values. Why were the dilutions stopped at that point, instead of continuing them to endpoint to allow the accurate calculation of SD50?

>>Although, several of the samples did not dilute all the way to zero seeding activity (i.e. 0/4 positive replicate wells), they each diluted to 2/4 or 1/4 positive wells, which is at or below the definition of 1 SD50, so the Spearman-Karber estimates of seed concentration should be very close to accurate, as we now explain in the Fig. 4 Legend (lines 176-181).

5. In the first paragraph of the Discussion, the authors should mention how the calculated seeding activity in the duodenal biopsies compares to seeding activity calculated using PD patient brain tissue and CSF. This would provide some context to the final statement in the Abstract ("the duodenum may be a source or a destination for pathological, self-propagating α-synuclein assemblies").

>>A good suggestion, which we have implemented on lines 245-247.

Reviewer #2: I only have a few minor comments to the discussion.

• Line 247 – 249: The reason for the increased sensitivity and specificity that we observed in the duodenum biopsies is unknown, but it could be due to the differences in the section of the intestine tested (rectum vs. duodenum) in the two patient cohorts and/or to the version of SAA used [18, 25]- > It has been shown that alpha-synuclein can spread from gut-to-brain and brain-to-gut via vagal pathways in animals models of body-first and brain-first PD (reviewed in PMID: 34952161). Since vagal innervation is highest in the upper gastroinstestinal tract, it would make sense to detect more aggregated alpha-synuclein in duodenal biopsies, compared to rectal biopsies. This should be included in the discussion.

>>We thank the reviewer for her insightful suggestion, and now bring up this possible explanation and added the reference that she mentioned on lines 254-257.

• Line 260-261: Thus, the link between the distribution of α-SynD in peripheral tissues and the stage of disease remains unclear [37]. --> Were the biopsies tested for inflammatory markers? Inflammation could temporarily upregulate the presence of asyn deposition, masking the correlation with clinical parameters. Such potential confounder should be discussed.

>>An interesting possibility. Unfortunately, we did not test for inflammatory markers, but raise this issue now on Lines 269-271.

• Line 292: While further studies are needed to elucidate the feasibility and utility of IM RT-QuICR, the potential for intra vitam monitoring and diagnosis, for example during routine esophagogastroduodenoscopy and colonoscopy screenings, holds promise for the early diagnosis of PD. --> The use of confirmation-specific oligothiophene ligands in combination with RT-QuICR should be included as future perspective for early stratification of synucleinopathies. Several labs have shown the power of using luminescent oligothiophenes (LCO) to differentiate asyn conformation between synucleinopathies (reviewed in PMID: 35693346). The LCO's have a much larger conformational freedom than ThT thanks to their flexible thiophene backbone, which allows conformation-specific binding to the aggregate. They yield a different spectral read out depending on the structure of the aggregate. The LCO staining could be done on a tissue section, or during the RT-QuIC procedure by replacing ThT with an LCO. Some labs have also used the LCO on the end-product of the RT-QuIC to investigate strain variability between end-product samples.

>>We thank the reviewer for this attractive suggestion for future development of RT-QuIC.

Reviewer #3: Minor Concern 1:
In Figure 1 the authors show tissue morphology but no evidence of α-synuclein within in the intestine. Although this may be difficult to detect it is important to show that pathologically there is α-synuclein within the tissues especially the mucosa region as this is what they claim within their studies to be important for RT-QuiCR screening.

>>Please see our response to this reasonable and natural request in our response to Reviewer #1.

Minor Concern 2:
The authors should discuss the rationale behind measuring the seeding only in the duodenum. Is there any advantages to this over the other portions of the intestine. Specifically the colon as most microbiome studies in the field are a snapshot of the intestinal tissue.

>>We had no particular rationale, other than the fact that duodenum biopsies were available from the PD patients in this study who underwent endoscopy to place a jejunal extension tube (PEG-J) for continuous levodopa enteral infusion. In this context, biopsies of other parts of the GI tract were not available.

Minor Concern 3:
As there is no correlation between clinical signs and the RT-QuiCR, how would one have this be a proper diagnostic? How does this limitation of your assay still allow for diagnostics? Important to discuss this in the discussion.

>>There is a strong correlation between overall clinical diagnosis of PD and IM RT-QuICR, hence our blinded determination that 22/23 PD IMs and 0/6 HC IMs were RT-QuICR positive. However, we have not seen significant correlation with the relative severity of those clinical signs. Thus, the assay should be helpful in diagnosing PD, but does not appear to be helpful in gauging disease stage or severity. We have raised this point on lines 263-268.

Minor Concern 4:
The authors find that there are specific parameters that must be used in order to get proper results from their seeding assay, mucosa from the 1st segment and no bilirubin contamination on the sample. This is critical and should be stated clearly in the discussion and a limitation of studying all routine colonoscopy procedures.

>>With respect, we are not sure where the reviewer got the impression that testing the 1st segment of the duodenum is crucial because, in 8/9 PD cases in which both segments were biopsied, both segments were positive (Fig 5). As for the suspected bilirubin effects (one sample only), we now mention the need for further study of this potentially confounding factor in the Discussion on lines 269-271.

Minor Concern 5:
The authors should discuss why they were unable to find tau in the intestine. Is it because its not there? The tool is not sensitive enough?

>>It is difficult to fully ascertain whether tau seeds are simply not there or are below our sensitivity to detect them. All we can say at this point is that tau RT-QuIC assay is extraordinarily sensitive with Alzheimer's brain tissue, as shown in Fig. 7, and when AD brain was spiked into dilutions of IM, we were able to detect a 100,000-fold dilution of AD brain.

PLOS authors have the option to publish the peer review history of their article (what does this mean?). If published, this will include your full peer review and any attached files.

If you choose "no", your identity will remain anonymous but your review may still be made public.

Do you want your identity to be public for this peer review? For information about this choice, including consent withdrawal, please see our Privacy Policy.

Reviewer #1: No

Reviewer #2: **Yes:** Nathalie Van Den Berge

Reviewer #3: **Yes:** Julie Moreno

Figure Files:

While revising your submission, please upload your figure files to the Preflight Analysis and Conversion Engine (PACE) digital diagnostic tool, https://pacev2.apexcovantage.com. PACE helps ensure that figures meet PLOS requirements. To use PACE, you must first register as a user. Then, login and navigate to the UPLOAD tab, where you will find detailed instructions on how to use the tool. If you encounter any issues or have any questions when using PACE, please email us at figures@plos.org.

>>Done.

Data Requirements:

Please note that, as a condition of publication, PLOS' data policy requires that you make available all data used to draw the conclusions outlined in your manuscript. Data must be deposited in an appropriate repository, included within the body of the manuscript, or uploaded as supporting information. This includes all numerical values that were used to generate graphs, histograms etc.. For an example see here: http://www.plosbiology.org/article/info%3Adoi%2F10.1371%2Fjournal.pbio.1001908#s5.

>>All relevant data have been included within the manuscript and uploaded as supporting information.

Reproducibility:

To enhance the reproducibility of your results, we recommend that you deposit your laboratory protocols in protocols.io, where a protocol can be assigned its own identifier (DOI) such that it can be cited independently in the future. Additionally, PLOS ONE offers an option to publish peer-reviewed clinical study protocols. Read more information on sharing protocols at https://plos.org/protocols?utm_medium=editorial-email&utm_source=authorletters&utm_campaign=protocols

>>We have provided what should be sufficient details to reproduce our results in the manuscript, but even more detailed αSyn RT-QuIC protocols have been (doi: 10.1007/978-1-4939-8820-4_2), and should soon be (Groveman et al., Meth. Mol. Biol., submitted), published elsewhere.

References:

Please review your reference list to ensure that it is complete and correct. If you have cited papers that have been retracted, please include the rationale for doing so in the manuscript text, or remove these references and replace them with relevant current references. Any changes to the reference list should be mentioned in the rebuttal letter that accompanies your revised manuscript. If you need to cite a retracted article, indicate the article's retracted status in the References list and also include a citation and full reference for the retraction notice.

>>Done.
